# Defensins: antifungal lessons from eukaryotes

**DOI:** 10.3389/fmicb.2014.00097

**Published:** 2014-03-20

**Authors:** Patrícia M. Silva, Sónia Gonçalves, Nuno C. Santos

**Affiliations:** Instituto de Medicina Molecular, Faculdade de Medicina, Universidade de LisboaLisbon, Portugal

**Keywords:** antimicrobial peptides, defensins, antifungal, resistance, host defense peptides

## Abstract

Over the last years, antimicrobial peptides (AMPs) have been the focus of intense research toward the finding of a viable alternative to current antifungal drugs. Defensins are one of the major families of AMPs and the most represented among all eukaryotic groups, providing an important first line of host defense against pathogenic microorganisms. Several of these cysteine-stabilized peptides present a relevant effect against fungi. Defensins are the AMPs with the broader distribution across all eukaryotic kingdoms, namely, Fungi, Plantae, and Animalia, and were recently shown to have an ancestor in a bacterial organism. As a part of the host defense, defensins act as an important vehicle of information between innate and adaptive immune system and have a role in immunomodulation. This multidimensionality represents a powerful host shield, hard for microorganisms to overcome using single approach resistance strategies. Pathogenic fungi resistance to conventional antimycotic drugs is becoming a major problem. Defensins, as other AMPs, have shown to be an effective alternative to the current antimycotic therapies, demonstrating potential as novel therapeutic agents or drug leads. In this review, we summarize the current knowledge on some eukaryotic defensins with antifungal action. An overview of the main targets in the fungal cell and the mechanism of action of these AMPs (namely, the selectivity for some fungal membrane components) are presented. Additionally, recent works on antifungal defensins structure, activity, and cytotoxicity are also reviewed.

## INTRODUCTION

Naturally occurring antimicrobial peptides (AMPs) probably represent one of the first successful forms of chemical defense of eukaryotic cells against bacteria, protozoa, fungi, and viruses (Ganz and Lehrer, 1998; Lehrer and Ganz, 1999; Zasloff, 2002; Mookherjee and Hancock, 2007; Lai and Gallo, 2009; Guo et al., 2012; Domingues et al., 2014), being also active against cancer cells (Hoskin and Ramamoorthy, 2008; Gaspar et al., 2013). Currently commercialized antibiotics are mostly of microbial origin or synthesized from those. These antibiotics are losing efficacy as a result of high selection pressure, leading to rapid emergence of resistance in many important human pathogens, thus threatening to put an end to the golden age of antibiotics (Clardy et al., 2009; Fisher et al., 2012). The use of antifungal treatments has increased as a consequence of the increase of immunocompromised patients, mostly due to improvements in oncology and transplant fields (Mehra et al., 2012), leading to more frequent resistances to the drugs used. A strategy to overcome this problem can be found in AMPs, which are part of the innate immune system of different living organisms (Hegedus and Marx, 2013), such as plants (Thomma et al., 2002; Lay and Anderson, 2005; Gonçalves et al., 2012b), fungi (Mygind et al., 2005; Schneider et al., 2010; Oeemig et al., 2012), bacteria (Zhu, 2007; Gao et al., 2009), invertebrates (Bulet and Stocklin, 2005; Ayroza et al., 2012), and vertebrates (Ganz, 2004; Sahl et al., 2005; van Dijk et al., 2008; Gonçalves et al., 2012a).

Although some AMPs have had their target unveiled, many are still unclear. Some mechanisms of action of antifungal peptides have been reported, such as binding to the cell wall, membrane permeabilization, receptor-mediated internalization inducing signaling cascades, and interaction with intracellular targets, inducing the formation of reactive oxygen species (ROS), leading ultimately to apoptosis (Hancock and Rozek, 2002; Oberparleiter et al., 2003; Thevissen et al., 2003a, 2004; de Coninck et al., 2013; van der Weerden et al., 2013). Apoptosis is a type of programed cell death, which is regulated by a complex network of proteins and metabolic pathways. The central core of this process is regulated by a family of proteins named caspases. Yeasts have at least one ortholog of mammalian caspases: the metacaspase YCA1 (yeast caspase 1; Madeo et al., 2002). Routinely used assays aiming at the detection of these apoptotic features are used for the identification of fungal cells undergoing apoptosis after treatment with antifungal agents.

Antimicrobial peptides have variable amino acid composition and size (ranging from less than 10 to more than 100 amino acid residues), commonly being cationic and amphipathic molecules (Brogden et al., 1996; Yang et al., 2003; Fontana et al., 2004; Glaser et al., 2005; Domingues et al., 2014). To date, more than 2200 natural or synthetic AMPs have been identified, as listed by the Antimicrobial Peptide Database (APD^1^), of which over 1900 have antibacterial activity and 800 have antifungal activity. This discrepancy, however, may be redundant as antibacterial AMPs may also have antifungal activity, but this property is not systematically assessed.

Antimicrobial peptides may have linear structures, like indolicidin (Ladokhin et al., 1999), or they may have tertiary structures stabilized by disulfide bonds, with β-sheet (e.g., protegrin; Aumelas et al., 1996; and the defensin human neutrophil peptide 1, HNP-1; Zhang et al., 2011), α-helix (e.g., dermaseptin; Mor et al., 1991) or αβ-motif secondary structure (e.g., drosomycin; Landon et al., 1997; and *Pisum sativum* defensin 1, *Ps*d1; Almeida et al., 2002).

The most studied families of AMPs are cathelicidins, dermaseptins, magainins, cecropins, and defensins. Cathelicidins are found in the innate immune system of mammals, amphibians, and reptiles (Wang et al., 2008; Tsai et al., 2011; Hao et al., 2012); dermaseptins and magainins are found in amphibians (Morton et al., 2007); cecropins are found in insects (YiZeng et al., 1989); and defensins, which are the largest family of AMPs, have also the broader distribution across the majority of eukaryotic organisms (Wang et al., 2013). Besides having antimicrobial activity, defensins also have immunomodulatory functions in the organisms that produce these peptides. Defensins with antifungal properties are present in all eukaryotic kingdoms, pointing out to a common ancestor. This review is focused on defensins (as well as some defensin-like peptides) with antifungal activity. However, it is impossible to describe here all the defensins with this activity. Therefore, we highlight some of the most recent research made on this field. The chosen peptides are described taking into consideration their specific properties, evolutionary background, organism of origin, and antifungal mode of action.

Some databases have been created in order to provide useful information for the study of AMPs. Among the AMP databases, PhytAMP is a database dedicated to antimicrobial plant peptides^2^ (Hammami et al., 2009). This resource contains valuable information on these AMPs, including peptide sequences, taxonomic, microbiological, and physicochemical data. Another database, Collection of Anti-Microbial Peptides (CAMP^3^), holds experimentally validated and predicted AMP sequences and structures of AMPs. These databases include several tools for AMPs analysis and prediction, helping in the design of new therapeutic peptides based on specific structure and functional features.

## DEFENSINS

Defensins are the largest groups of AMPs. These peptides are cysteine-rich and have diverse sequences and structures, stabilized into compact shapes by three or four conserved cysteine disulfide bridges. They have at least two positive charges (lysine or arginine residues) and are small, ranging approximately from 12 to 50 amino acid residues (2–6 kDa; Ganz, 2003; Ren et al., 2011; Gao and Zhu, 2012).

**FIGURE 1:**
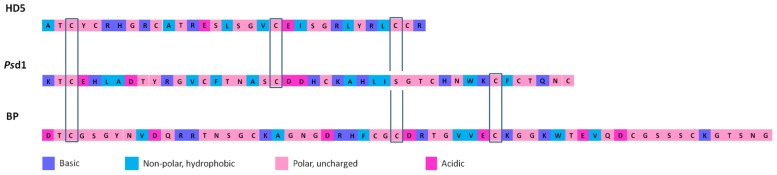
**Comparison of amino acid residues sequence of selected defensins with antifungal activity from animals, plants, and fungi: human defensin 5 (HD5), *Pisum sativum* defensin 1 (Psd1), and *Penicillium brevicompactum* Dierckx bubble protein (BP), respectively.** Conserved cysteine residues among defensins from different kingdoms are indicated. The colors used in the sequences represent basic, non-polar hydrophobic, polar-uncharged and acidic residues.

Vertebrates’ defensins are divided into three subfamilies: α-, β-, and θ-defensins. α-Defensins are present in mammals such as humans, monkeys, and several rodent species, being particularly abundant in neutrophils, certain macrophage subpopulations and Paneth cells of the small intestine (Ouellette and Selsted, 1996; Ganz and Lehrer, 1998; Lehrer and Ganz, 1999). β-defensins are found in a wide range of vertebrates, presenting a cysteine-stabilized αβ-motif composed of an antiparallel β-sheet and an α-helix. As an example, on bovine neutrophils, as many as 13 β-defensins have been identified (Yang et al., 2002a). However, in other species, β-defensins are mostly produced by epithelial cells lining different organs (e.g., epidermis, bronchial tree, and genitourinary tract). θ-Defensins, present only in Old World monkeys, are cyclic and derived from α-defensins (Lehrer, 2004; Lehrer and Lu, 2011; Semple and Dorin, 2012). In **Figure 1**, conserved cysteine residues among defensins from different kingdoms are shown.

Plants, fungi, and many invertebrates produce defensin-like peptides structurally similar to the β-defensins from vertebrates (Thomma et al., 2002; Bulet and Stocklin, 2005; Mygind et al., 2005; Sahl et al., 2005; Yount and Yeaman, 2006; van Dijk et al., 2008; Ayroza et al., 2012; Oeemig et al., 2012). These observations allowed to assume that defensins and defensin-like peptides all evolved from a common precursor. The relatively recent identification of three defensins in lower eukaryotes, plectasin from *Pseudoplectania nigrella* (Mygind et al., 2005), eurocin from *Eurotium amstelodami* (Oeemig et al., 2012), and bubble protein (BP) from *Penicillium brevicompactum* Dierckx (Seibold et al., 2011), is important to demonstrate the wide distribution of these peptides over diverse eukaryotic lineages, which suggests that ancestral defensin genes existed over 1500 million years ago, before Fungi, Plantae, and Animalia kingdoms diverged (Wang et al., 1999). The wide distribution of these peptides in the Eukarya domain could suggest their uniqueness to eukaryotic cells, but it was possible to determine that these peptides may have had their ancestor in a prokaryotic organism after the discovery of the first defensin-like peptide in the bacteria *Anaeromyxobacter dehalogenans* (AdDLP; Zhu, 2007; Gao et al., 2009). This defensin-like peptide is proposed as an ancestor of eukaryotic defensins and defensin-like peptides due to the similarity of their structures, namely at the level of the cysteine-stabilized αβ-motif (Zhu, 2007). These findings further support the concept that AMPs may have been fundamental to the evolution of multicellular organisms within microbial-exposed environments.

Although defensins were initially identified only as AMPs, recent studies have demonstrated that they have a much broader range of action, including immunomodulatory function (issue further developed in the text, in Section “Immunomodulatory Function”; Ulm et al., 2012).

## RECENTLY STUDIED ANTIFUNGAL DEFENSINS

When a new AMP is described, the most usual properties to assess are structure and peptide sequence, antimicrobial activity, expressed mainly in terms of minimal inhibitory concentration (MIC) or half maximal inhibitory concentration (IC_50_), cytotoxicity and lytic activity against human cells (whenever the AMP origin is not mammalian), target, and mode of action toward the pathogen tested. The following AMPs were classified as defensins or defensin-like peptides due to their structural and sequence homologies with other defensins. They are examples of some of the recently studied antifungal defensins from fungal, plant, and animal origin that fulfill most of the properties expressed above. Further details and a list of some of these defensins can be found in **Table 1**.

**Table 1:** Recently studied antifungal defensins from fungal, plant, and animal sources.

AMP	Organism of origin	Antifungal spectrum	Antifungal activity	Mode of action	Cytotoxicity	Reference
**Fungi**
PAF	*Penicillium chrysogenum*	Several species of Zygomycetes, Ascomycetes, and Basidiomycota	MIC: 1–200 μg/ml	Interaction with G protein signal transduction pathways, leading to production of ROS and induction of apoptosis	Non-toxic to mammalian cells	Kaiserer et al. (2003); Marx (2004); Galgóczy etal. (2005,^2007^,2008); Barna etal. (2008)
AFP	*Aspergillus giganteus*	Several species of Ascomycetes**	MIC: 1–200 μg/ml	Specific inhibition of the chitin synthase III and V, interfering with cell wall biosynthesis	Non-toxic to mammalian cells	Lacadena et al. (1995);Moreno et al. (2003); Moreno et al. (2006); Theis et al. (2003); Meyer (2008)
Bubble protein	*Penicillium brevicompactum* Dierckx	*Saccharomyces cerevisiae*	Growth inhibition in a dose-dependent manner**	n.d.**	n.d.**	Olsen et al. (2004); Seibold et al. (2011)
**Plant**
*Ps*d1	Pea (*Pisum sativum*)**	*Aspergillus* spp.; *Fusarium solani*; *Neurospora crassa*; *Candida albicans*	IC_50_: 0.04–21.7 μg/ml; MIC *C. albicans*: 20 μM	Impairment of progression of cell cycle: cyclin F; S to G2 phase transition is blocked, resulting in endoreduplication; strong interaction with ergosterol and fungal sterol-rich membranes	Reduced interaction with cholesterol-rich mammalian membranes	Almeida et al. (2000); Lobo et al. (2007); de Medeiros (2009); de Medeiros et al. (2010); Goncc (2012b)
*Rs*AFP2	Radish (*Raphanus sativus*)**	*Candida* spp.; *Aspergillus flavus*; *Fusarium solani*	IC_30_ *C. albicans*: 10 μg/ml. Reduced cell viability in all *Candida* spp. tested with up to 10 μM of RsAFP2	Interaction with glucosylceramides; membrane permeabilization; ROS production; cell growth arrest; apoptosis induction; caspase activation; yeast-to-hypha transition blocking; septin localization; ceramide accumulation; altered cell wall shape	Non-toxic to mammalian cells	Aerts etal. (2007,2009); Tavares et al. (2008); Thevissen et al. (2012)
*Hs*AFP1	Coral bells (*Heuchera sanguinea*)**	*Neurospora crassa*; *Candida albicans*	IC_50_ *Neurospora crassa*: 4 μg/ml	Interaction with cell membrane (hypha); ROS formation; apoptosis induction	n.d.	Thevissen et al. (1997); Aerts et al. (2011)
**Animal**
**Arthropod**
Coprisin	Korean dung beetle (*Copris tripartitus*)	*Aspergillus* spp.; *Candida* spp.; *Malassezia furfur*; *Trichosporon beigelii*; *Trichophyton rubrum*	MIC: 5–20 μM	Apoptosis induction; ROS formation; disruption of mitochondrial membrane potential; cytochrome *c* release; intracellular metacaspase activation	No hemolytic activity on human erythrocytes	Lee etal. (2012)
Juruin	Amazonian pink toe spider (*Avicularia juruensis*)**	*Candida* spp.; *Aspergillus niger*	MIC: 2.5–10 μM; fungicidal activity, rather than fungistatic	n.d.	No hemolytic activity on human erythrocytes	Ayroza etal. (2012)
**Reptile**
Crotamine	South-American rattlesnake (*Crotalus durissus terrificus*)	*Candida* spp.; *Trichosporon* spp.; *Cryptococcus neoformans*	MIC: 12.5–50 μg/ml; fungicidal activity, rather than fungistatic	Pronounced ultrastructural alterations; membrane collapse; cytoplasmic coagulation	No hemolytic activity on human erythrocytes; CC_50_ > 50 μM against non-tumoral animal and human cells	Yamane etal. (2013)

### FUNGAL SOURCES

Defensin-like antifungal peptides secreted by filamentous fungi have a low molecular mass (5.8–6.6 kDa), a basic character, presence of 4–10 cysteine residues and several disulfide bonds (providing resistance against temperature stress or adverse solvent conditions), and a β-barrel conformation (Hagen et al., 2007; Seibold et al., 2011). Proteins with such properties with antifungal activity have been isolated and investigated from several Ascomycota fungal species, such as *Penicillium chrysogenum*, *Penicillium nalgiovense*, *Penicillium brevicompactum* Dierckx, *Aspergillus giganteus*, and *Aspergillus niger* (Galgóczy et al., 2010; Seibold et al., 2011). Among these fungus-derived antifungal peptides, the most intensively studied are *Penicillium chrysogenum* antifungal protein (PAF) and *Aspergillus giganteus* antifungal protein (AFP), with six and eight cysteines, respectively. *Penicillium brevicompactum* Dierckx bubble protein structure is considerably similar to PAF and AFP.

#### Penicillium chrysogenum *antifungal protein*

PAF acts through a G protein-coupled signal transduction pathway, although this mechanism is not entirely understood (Marx et al., 2008). The G protein-coupled activity of PAF was confirmed by the study of the *fadA* mutant of *Aspergillus nidulans* which proved to be less sensitive to PAF treatment compared to the wild-type. The *fadA* gene encodes the heterotrimeric G protein α subunit, which dissociation from the G_β__γ_ complex is inhibited in the *fadA* mutant *Aspergillus nidulans* (Leiter et al., 2005). These results indicate that PAF toxicity requires active heterotrimeric G protein signaling (Leiter et al., 2005; Marx et al., 2008). Based on these observations, Marx et al. (2008) hypothesized that PAF interacts directly or indirectly with G protein signal transduction pathways. These authors also hypothesized that the cell wall could be a selective barrier for PAF, but *in vivo* chitin-binding activity of PAF has not been demonstrated yet. In the sensitive organisms, PAF exerts multiple detrimental effects: induction of plasma membrane polarization, increased exposure of phosphatidylserine (PS) on the cell surface, DNA fragmentation, membrane blebbing, cell shrinking, intracellular ROS production, and apoptosis-like phenotype (Kaiserer et al., 2003; Leiter et al., 2005; Marx et al., 2008).

#### Aspergillus giganteus *antifungal protein*

In susceptible organisms, AFP disturbs the polarized growth of hyphae by interfering directly or indirectly with the cell wall biosynthesis (Theis et al., 2003). Hagen et al. (2007) demonstrated that AFP can bind to chitin *in vitro*, and inhibits the cell wall chitin biosynthesis by the specific inhibition of chitin synthase III and V. These enzymes are unique among fungi and essential for the maintenance of the polarized growth and virulence of pathogenic fungi. Presence of chitin synthase III and V is confirmed in the AFP-sensitive fungi, while the insensitive fungal species do not have these enzyme classes. Sphingolipid membrane components in the sensitive fungi might serve as secondary receptors for AFP. This was further confirmed by the discovery that the depletion of cellular glucosylceramide (GlcCer) levels in AFP-sensitive fungal species (*Aspergillus fumigatus* and *Aspergillus niger*) resulted in reduced AFP susceptibility (Meyer, 2008). This, together with the observation that the sphingolipids are necessary to maintain the polarized hyphal growth, elucidates the mechanism of polarized growth degeneration effect of AFP (Li et al., 2006; Meyer, 2008). The species specificity of AFP may be related with the sphingolipid profile of the sensitive fungi (Meyer, 2008).

#### Penicillium brevicompactum *Dierckx bubble protein*

This fungal defensin was first described in 2003 (Olsen et al., 2004). It is found in the bright yellow–green fluorescent exudate bubbles of the ascomycete fungus *Penicillium brevicompactum* Dierckx. Similarly to other ascomycetes, BP produces a small antimicrobial molecule, mycophenolic acid, which gives the bubbles their yellow–green fluorescence. This combined production suggests a possible synergistic action between defensins and other antibiotic agents produced by this class of fungi (Seibold et al., 2011). BP has 64 amino acid residues, with high content of basic amino acids, β-barrel conformation (Olsen et al., 2004), and a cage-like pattern of four very stable disulfide bridges. In addition, it was discovered that the closely related fungus *Penicillium chrysogenum* encodes a BP homolog (in addition to PAF), indicating that fungi may have more than one defensin (Seibold et al., 2011).

### PLANT SOURCES

Many fungi are phytopathogenic, with species such as *Fusarium* spp., *Cladosporium* spp., *Pythium* spp., *Curvularia* spp., *Aspergillus flavus*, and *Puccinia pittieriana* affecting potato, rice, corn, wheat, tobacco, and cotton crops by causing wilt, mold, crown rot, mildew, and rust, just to name a few plant diseases (The American Phytopathological Society, APS^4^). These diseases can deplete entire crops, bearing enormous costs for agriculture due to the difficulty in eliminating fungal infections from plants, once they appear. Soils harbor plants for most of their life cycle, but also a considerable amount of bacteria, fungi, and parasites, many of which can be phytopathogenic. For this reason, plants need to have good defenses against these microorganisms; thus, it is easy conceivable that plants are major AMPs producers, often with antifungal activity, but also antibacterial activity (Moreno et al., 1994; Segura et al., 1998; Thomma et al., 2002; Mayer et al., 2013).

In fact, a major research effort has been put forward on the screening for these molecules in plants. Besides defensins, other AMPs are also produced by plants, being exclusive to them. Examples of these plant exclusive AMPs are thionins, lipid transfer proteins and snakins, which were also demonstrated to have antifungal activity (Segura et al., 1999; Silverstein et al., 2007; Sun et al., 2008; Asano et al., 2013). Plant defensins with antifungal activity have been purified from several plants, such as *Pisum sativum* (Almeida et al., 2000; Lobo et al., 2007; de Medeiros, 2009; de Medeiros et al., 2010; Gonçalves et al., 2012b), *Raphanus sativus* (Aerts et al., 2007, 2009; Tavares et al., 2008; Thevissen et al., 2012), and *Heuchera sanguinea* (Thevissen et al., 1997; Aerts et al., 2011), which will be addressed below. Several other plant defensins with antifungal activity have also been studied. Specific information about some of those defensins can be found on the following references: *Medicago sativa* defensin 1 (*Ms*Def1) and *Medicago truncatula* defensin 4 (*Mt*Def4; Spelbrink et al., 2004; Ramamoorthy et al., 2007a,b; Sagaram et al., 2011); *Dahlia merckii* AMP 1 (*Dm*AMP1; Thevissen et al., 1999, 2000a,b, 2003b; Jha et al., 2009; Salahinejad et al., 2013); *Phaseolus vulgaris* defensin 1 (*Pv*D1; Games et al., 2008; Mello et al., 2011; Wu et al., 2011; Chan et al., 2012; Wong et al., 2012; Chan and Ng, 2013); *Nicotiana alata* defensin 1 (*Na*D1; Lay et al., 2003, 2012; van der Weerden et al., 2008, 2010; Hayes et al., 2013).

#### Ps*d1*

This garden pea (*Pisum sativum*) seed defensin, firstly characterized in 2000 (Almeida et al., 2000), has 46 amino acid residues. Its secondary structure comprises a globular fold composed of β-sheets and an α-helix stabilized by four disulfide bridges, i.e., a cysteine-stabilized αβ-motif (Almeida et al., 2002). As demonstrated by a yeast two-hybrid screening system, *Ps*d1 has affinity to a *Neurospora crassa* protein related to the cell cycle control, cyclin F (Lobo et al., 2007). Using a developing retinal tissue of neonatal rats as a model to study this interaction, it was proven that *Ps*d1 impairs the correct cell cycle progression, by blocking cyclin F role in the transition of S to G2 phases of the cell cycle, promoting endoreduplication and disturbing nuclear migration. Recently, it has been demonstrated through partition studies that *Ps*d1 has a high affinity with high specificity to model membranes enriched with ergosterol, the main sterol present in fungal membranes, and GlcCer (Gonçalves et al., 2012b). On the contrary, there is no interaction between the defensin and model membranes enriched in cholesterol (a characteristic of mammalian cells), reducing *Ps*d1 toxicity to human cells.

#### Rs*AFP2*

This defensin, isolated from radish (*Raphanus sativus*) seeds in 1992 (Terras et al., 1992), has 51 amino acid residues and is highly cationic. It has eight cysteine residues, forming four disulfide bridges that stabilize its αβ-motif structure. *Rs*AFP2 has fungal GlcCer as its target, as observed in experiments performed with wild-type *Candida albicans* and a mutant lacking GlcCer in the membrane (Δ*gcs*; Aerts et al., 2007). It does not need to be internalized to have its antifungal effect. After the initial contact, a signaling cascade is activated inside the cell and ROS are formed, leading to membrane permeabilization and consequent cell death (Aerts et al., 2007). Other effects of *Rs*AFP2 comprise the induction of apoptosis in *C. albicans* by triggering caspases activation, but not of metacaspases, implying that different apoptotic pathways can be induced in *C. albicans* (Aerts et al., 2009). *Rs*AFP2 also promotes an accumulation of ceramides in *C. albicans*, which can be lethal to the cell, and blocks the yeast-to-hypha transition (Thevissen et al., 2012). *In vivo* experiments were performed in murine models, proving that *Rs*AFP2 considerably reduces the fungal burden in kidneys of mice infected with *C. albicans*. This defensin has low susceptibility to serum peptidases, meaning that upon entering the bloodstream it will not be degraded, maintaining its antifungal activity. Lactate dehydrogenase (LDH) release levels are indicative of cell damage and tissue breakdown. Human brain endothelial cells incubated with *Rs*AFP2 show no release of LDH, hence supporting the conclusion that this defensin has limited toxicity to mammalian cells.

#### Hs*AFP1*

Firstly identified in 1995, this defensin found in the seeds of coral bells plant *H. sanguinea* (Osborn et al., 1995) was shown to have high affinity to specific sites in fungal membranes and to permeabilize cells of susceptible fungi (Thevissen et al., 1997). Unlike *Rs*AFP2, which relies on an interaction with GlcCer to exert its antifungal effect, *Hs*AFP1 has antifungal activity against *C. albicans* Δ*gcs* and its wild-type counterpart (Aerts et al., 2011). It was proposed that *Hs*AFP1 may interact with essential components of the fungal membrane, resulting in a low occurrence of resistance *in vitro*, an advantage for the use of *Hs*AFP1 as a novel antifungal agent (Aerts et al., 2011). Using sodium azide, a respiratory inhibitor, mitochondrial function is impaired and *Hs*AFP1 antifungal activity is affected, indicating that the defensin requires a properly working respiratory chain. This defensin induces ROS formation and apoptosis in yeast. It was also proposed that mitogen-activated protein kinase (MAPK) signaling pathways may be a possible strategy for yeast tolerance to *Hs*AFP1 (Aerts et al., 2011).

### Animal Sources

#### Mammal defensins

Antimicrobial peptides from animal sources have shown antifungal and immunomodulatory activities, being mammals major producers of defensins (Yang et al., 2002a,b; Sahl et al., 2005). θ-Defensins are the less studied defensin family, at least partially due to their source. To date, no antifungal activity was attributed to θ-defensins; as such, these defensins will not be further discussed in the present review. Being vertebrates, mammals possess an adaptive immune system, hence having a more complex network of signaling pathways, diverse responses against pathogens invading the organism and an array of AMPs produced in different organs and tissues, each with its particular function and mode of action (Ganz and Lehrer, 1998; Ganz, 2004; Lai and Gallo, 2009; Pasupuleti et al., 2012). Human β-defensins 1 and 2 are chemotactic for memory T cells and immature dendritic cells (Pazgier et al., 2006). Mammal defensins differ substantially in their antimicrobial specificities. For example, HNP-1, HNP-5 and human beta-defensins 1 and 3 (HBD1 and HBD3, respectively) have broad antimicrobial activities against Gram-negative and Gram-positive bacteria and yeasts (Ganz et al., 1985; Bensch et al., 1995; Porter et al., 1997; Harder et al., 2001; Hoover et al., 2003; Joly et al., 2004). HBD1 and HBD3 have been shown to be effective against *C. albicans* (Krishnakumari et al., 2009), while HBD2 has been shown to possess significant microbicidal activity against Gram-negative bacteria and *C. albicans* (Schroder and Harder, 1999). Recombinant human intestinal defensin 5 (rHD-5) exhibits microbicidal activity against *Listeria monocytogenes*, *Escherichia coli*, and *C. albicans*. Opposed to cryptdins, the mouse intestinal defensins, rHD-5 is active against both mouse-virulent wild-type *Salmonella typhimurium* and its isogenic, mouse-avirulent *pho*P mutant (Porter et al., 1997).

Mouse β-defensin 3 (MBD3), a HBD2 homolog, is an AMP expressed in the mouse epithelial and mucosal tissues (Jiang et al., 2010). The fungicidal properties of recombinant MBD3 suggest that similar peptide formulations can be used in the treatment of fungal and/or bacterial infections. MBD3 is expressed in footpads, skin, and mucosal membranes (tongue) of normal mice. Potent antifungal activity was observed against filamentous fungi, such as *Aspergillus fumaricus*, *Microsporum canis*, *Trichophyton rubrum*, *Trichophyton tonsurans*, and *Trichophyton violaceum* (all these species are primary human pathogens, meaning they cause infection whether or not the immune defenses are weakened, as opposed to opportunistic fungal infections), as well as yeast strains like *C. albicans* and *Cryptococcus neoformans*. This peptide also presents bactericidal activity against Gram-positive and Gram-negative bacteria, such as *Staphylococcus aureus*, *E. coli*, and *Salmonella typhi* (Jiang et al., 2010).

#### Arthropods defensins

***Coprisin***. This 43 amino acid residues beetle defensin-like peptide was described in 2009 as an antibacterial peptide (Hwang et al., 2009). Its structure comprises an αβ-motif, stabilized by three cysteine disulfide bridges (Lee et al., 2013). In 2012, the same authors investigated its antifungal activity against *C. albicans*, revealing that coprisin enters the fungal cell and localizes in the nucleus, which indicates that coprisin penetrated the membrane without disrupting the fungal plasma membrane, as confirmed with 1,6-diphenyl-1,3,5-hexatriene (DPH) analysis, calcein-leakage, and giant unilamellar vesicle assays (Lee et al., 2012). Using H_2_O_2_ as a positive control for apoptotic induction, coprisin proved to have the same effects in inducing early and late apoptosis, features shown by the annexin V conjugated with fluorescein and propidium iodide co-staining method. Apoptosis induced by this AMP is metacaspase-dependent. Concomitantly, coprisin compromises mitochondrial membrane potential and ROS production, in addition to the release of cytochrome *c* from the mitochondria to the cytosol. No hemolytic activity was observed for this peptide in human erythrocytes (Lee et al., 2012).

***Juruin***. This defensin-like peptide was discovered in 2012 by screening the venom on the theraposid Amazonian pink toe spider *Avicularia juruensis* (Ayroza et al., 2012). It has 38 amino acid residues, three disulfide bonds and, like neurotoxins reported to have antimicrobial activity, it has a putative inhibitory cysteine knot (ICK) motif, i.e., a fold common to venom peptides from spiders, scorpions, and aquatic cone snails (Smith et al., 2011). ICK-containing peptides of spider venom are likely to have evolved from β-defensins (Fry et al., 2009). Based on amino acid sequence and structure similarities with insecticidal peptides of other spiders, this peptide is likely to belong to a group of conserved toxins with voltage-gated ion channels inhibitory action. Juruin showed a fungicidal rather than fungistatic effect against *C. albicans* and *C. tropicalis*, without hemolytic activity (Fry et al., 2009).

#### Reptile defensins

***Crotamine***. This highly basic peptide, isolated from the venom of a South-American rattlesnake, was discovered in 1947 (Gonçalves and Polson, 1947). It shares structural similarity to β-defensins due to an identical disulfide bridge pattern (Fadel et al., 2005; Yamane et al., 2013). Crotamine structure comprises an antiparallel β-sheet and an α-helix stabilized by three disulfide bridges (Nicastro et al., 2003; Fadel et al., 2005). Recombinant crotamine displayed a more potent antimicrobial activity than native and synthesized crotamine (Yamane et al., 2013). This peptide induces extensive ultrastructural modifications in *C. albicans*. TEM studies showed deformed cell shape, irregular layering structure of cell wall and cytoplasmic contents coagulation, but without detectable hemolytic effects and low toxicity to mammalian cells (Yamane et al., 2013).

## IMMUNOMODULATORY FUNCTION

Defensins may be produced constitutively or have their expression triggered when there is an inflammatory process, by the recognition of microbial conserved structures, such as lipopolysaccharide (LPS) and lipoteichoic acid, or inflammatory effectors, like cytokines. These AMPs are expressed differentially depending on the peptide itself and on the tissue or cell type (Ulm et al., 2012). Defensins, besides their antimicrobial action, can also be immunomodulatory and inhibitors of virulence factors. This ability is not exclusive of defensins, as other AMPs also share this property. Thus, they can enhance the host’s immune system, with this multifunctional character rendering these peptides lower probability of becoming tolerated by microorganisms (Mehra et al., 2012; Jarczak et al., 2013).

Pro-inflammatory mediation has been recognized in some of these molecules, as they can bind to chemokine receptors, being able to recruit immune cells, thus enhancing the immune response (Mookherjee and Hancock, 2007; Lai and Gallo, 2009; Alba et al., 2012; Semple and Dorin, 2012; Ulm et al., 2012; Zhu and Gao, 2013). β-Defensins were demonstrated to have the capability to induce chemoattraction of CD4^+^ memory T cells, macrophages, and immature dendritic cells, by binding to receptors in the membrane (Yang et al., 1999; Wu et al., 2003; Taylor et al., 2008). This binding favors the attraction and migration of inflammatory cells to the inflammation site, in order to improve and speed up the inflammatory response. α and β-Defensins have also been shown to inhibit neutrophil apoptosis (Nagaoka et al., 2008). These authors showed that HBD3 binds to CCR6 at the neutrophil cell surface, initiating an increase in the levels of the antiapoptotic protein Bcl-xL and inhibiting caspase activity. This increases neutrophils life span and is an inflammatory event that is beneficial to eradicate invading microorganisms (Nagaoka et al., 2008), thus promoting the production of proinflammatory cytokines and chemokines, which in turn, amplifies the immune system response. Defensins have been shown to have a proinflammatory effect on human keratinocytes (Niyonsaba et al., 2007). Treatment of these cells with HBD2 HBD3 or HBD4 leads to the increase of the expression of pro-inflammatory mediators, like monocyte chemoattractant protein-1, macrophage inflammatory protein-3, and some interleukins (Niyonsaba et al., 2007).

Surprisingly, some defensins are also able to attenuate pro-inflammatory responses whenever these can be harmful to the organism (Lande et al., 2007; Yamasaki et al., 2007). These antagonistic effects depend on the level of expression, disease state, and pathogen exposure. It has been previously described for α-defensins that mice having a matrilysin deficiency (hence without mature α-defensins in the intestine) are more susceptible to chemically induced colitis than wild-type controls. Interleukin-1β (IL-1β), a cytokine with an important role in mediation of inflammation, reaches level significantly increased in the deficient mice and it was ultimately shown that α-defensins are able to inhibit the production of IL-1β (Shi et al., 2007).

It has been demonstrated that HBD3 (mainly expressed in epithelial cells), when in basal concentration, has an immunosuppressive effect in the presence of LPS, contributing to the maintenance of a non-inflammatory environment over continual low-level exposure to microorganisms, commensal or pathogenic (Semple et al., 2010). Concentrations of HBD3 ranging from 0.5 to 1 μM are able to suppress the induction of tumor necrosis factor α (TNFα), a proinflammatory effector of the immune system, and IL-6, an interleukin that acts both as pro and anti-inflammatory. At these concentrations, proinflammatory proteins are not induced and there is no proinflammatory gene expression (Semple et al., 2010). The proinflammatory effects of β-defensins were observed at slightly higher concentrations of the defensin, in the 4–6 μM range (Funderburg et al., 2007; Niyonsaba et al., 2010). This was not the first case observed of opposite effects in immunomodulating AMPs. Cathelicidin LL-37 has been shown to have also a duality in inflammatory effects, being proinflammatory at concentrations above 20 μg/ml but anti-inflammatory at 1–5 μg/ml (Scott et al., 2002). Defensins were also shown to have a role in other biological processes, namely wound healing (Hirsch et al., 2009), dog coat color determination (Candille et al., 2007), fertility (Li et al., 2001), plant development (Stotz et al., 2009), and carcinogenesis regulation (Donald et al., 2003; Gambichler et al., 2006; Joly et al., 2009).

It is clear that defensins have many functions that are determined by the level of expression. Whereas higher expression of defensins takes place at the pathogen’s site of entry, with a proinflammatory response and the chemoattraction of macrophages and other immune cells, defensins expressed at lower levels may be involved in the resolution of the immune response. When the danger is neutralized and defensins and other proinflammatory molecules decrease in the inflammation site, defensins may then have a role in resolving inflammation (Semple and Dorin, 2012).

Due to this multifunctionality, AMPs have also been referred to as host defense peptides (HDPs; Steinstraesser et al., 2010; Ulm et al., 2012).

## RESISTANCE

Like other antibiotics resistance, it is easily conceivable that AMPs resistance is a key characteristic for increased virulence of pathogenic strains. Despite this fact, and contrary to antibiotics that act through a single approach (meaning that microbes can evade them through a single resistance system), AMPs follow a multidimensional strategy against microbial invasion (Lai and Gallo, 2009). Therefore, selective pressures on microbes are avoided, reducing the development of resistant strains (Zasloff, 2002).

A synergistic effect between different host AMPs is also possible, as evidenced by the fact that the MIC of AMPs *in vitro* are usually higher than the physiological concentrations of those AMPs *in vivo* (Lai and Gallo, 2009). Two distinct AMPs may have their combined MIC much lower than when acting isolated, strongly suggesting heterologous HDP interactions (Westerhoff et al., 1995).

Microorganisms have evolved their own strategies for evading the antimicrobial action of the compounds used against them. AMPs frequently have the ability to disrupt microbial membranes and to inhibit the synthesis of some of their components; thus, strategies to escape the action of those AMPs follow the redesign of cell membranes, as described for both Gram-negative and Gram-positive bacteria (Gunn et al., 2000; Li et al., 2007). Other evasion mechanisms include affecting the correct function of the AMP by turning off its expression, releasing plasmid DNA in epithelial cells, a strategy adopted by highly contagious bacteria from the *Shigella* genus that cause dysentery (Islam et al., 2001). As AMPs frequently rely on transmembrane potential to interact with microbial pathogens and exert their mechanism of action against them, it is probable that another microbe strategy for evading AMPs could be to change their transmembrane potential status (Yeaman and Yount, 2003).

*Candida albicans* resistance to some AMPs is regulated by the protein Ssd1, combined with the transcription factor Bcr1 (biofilm and cell wall regulator; Nobile and Mitchell, 2005; Gank et al., 2008; Jung et al., 2013). Ssd1 is an RNA-binding protein and a component of the regulation of morphogenesis pathway (Saputo et al., 2012). In *C. albicans*, this pathway governs multiple processes, including filamentation and cell wall integrity (Song et al., 2008; Bharucha et al., 2011). This combination yields resistance to protamine, RP-1 and HBD2 by maintaining mitochondrial energetics and reducing membrane permeabilization, thus allowing the fungus to counteract the negative effects of these AMPs (Jung et al., 2013). Protamine is an α-helical cationic polypeptide, frequently used to screen for AMP susceptibility (Yeaman et al., 1996), and RP-1 is a synthetic AMP modeled upon the C-terminal α-helical domain existent in the human platelet factor-4 kinocidins; this domain is responsible for RP-1 microbicidal activity (Bourbigot et al., 2009). *C. albicans* mutant strains in Bcr1 and Ssd1 proteins are more susceptible to the AMPs described above; thus, Jung et al. (2013) were able to conclude that these proteins are necessary for the resistance to protamine, RP-1 and HBD2. Further studies are necessary to clarify the roles of Bcr1 and Ssd1 in early *versus* late mechanisms of resistance to AMPs.

The Hog1 (high osmolarity glycerol) MAPK pathway, which provides a response to osmotic, oxidative, and heavy-metal exposure stresses in fungal cells, was shown to be activated in the presence of AMPs, such as *Na*D1, HBD2, HBD3, and histatin-5 (a salivary cationic AMP that has a role in keeping *C. albicans* in its commensal state; Yeaman et al., 1996; Vylkova et al., 2007; Argimon et al., 2011; Hayes et al., 2013). The injuries imposed on *C. albicans* by these defensins seem to share common features with osmotic and/or oxidative stress (Argimon et al., 2011). Upon exposure to these defensins, the Hog1 MAP kinase is activated, triggering a transcriptional response aimed to rescue the cells from the source of injury, i.e., the core and osmotic-stress transcriptional responses (Enjalbert et al., 2006; Argimon et al., 2011).

Another strategy for evading AMP function is to enzymatically degrade these peptides before they exert their effects. This is possible by producing proteases and peptidases involved in tissue degradation, as described for *C. albicans* secreted aspartic proteases (Saps). Namely, histatin-5, present in human saliva, is a host-specific substrate of Sap9, enabling the transition of the fungus from commensal to pathogenic in HIV^+^ individuals. These patients, who have lower levels of this isoenzyme in the saliva, have an increased incidence of oral candidiasis (Meiller et al., 2009; Khan et al., 2013). Also regarding histatin-5, a transport mechanism of efflux mediated by the flu-1 transporter has been described for *C. albicans*, rendering the pathogen the ability to reduce the isoenzyme cytosolic concentration and fungicidal activity (Li et al., 2013). The LL-37 cathelicidin and histatins bind to cell wall carbohydrates, preventing adhesion of *C. albicans* to host cells; thus, the release of AMP-binding proteins acts as a decoy for these AMPs, diverting them from binding to fungal cell surface (den Hertog et al., 2005, 2006; Mochon and Liu, 2008). For example, Msb2 (multicopy suppressor of a budding defect) is a *C. albicans* surface protein (a mucin) highly soluble and proteolytically stable, which is shed to the extracellular environment, acting as a basal AMP-resistance decoy by binding to LL-37 and histatin-5, avoiding the antimicrobial action of these AMPs (Szafranski-Schneider et al., 2012).

The characteristics described above are associated with a decrease in microbes’ susceptibility to AMPs, indicating that microbial pathogens have developed some structure-specific and energy-dependent mechanisms to subvert the action of these host defense systems.

## FUNGAL CELL MEMBRANE

Fungi possess a unique cell wall and cell membrane that can serve as specific targets for antifungal agents. The fungal cell membrane is similar to those of other eukaryotic cells, composed of a lipid bilayer with proteins embedded within it (Katzung et al., 2011). Sterols (absent in prokaryotes) are major components of fungal membranes. The sterol present in higher eukaryotic membranes is cholesterol, but in fungal membranes the main sterol present is ergosterol, providing stability and flexibility to the cell membrane (Thevissen et al., 1999, 2003a).

Glycosphingolipids (GSLs) are a family of lipids that act as key components of biological membranes. They exist in animals, plants, and fungi (Leipelt et al., 2001; Halter et al., 2007; Daniotti and Iglesias-Bartolome, 2011). GSLs were initially described as components of the architecture of cell membranes, straightly connected with fluidity and stability (Feinstein et al., 1975; Aaronson and Martin, 1983; Campanella, 1992). Recently, however, it was demonstrated that their role goes clearly beyond the initial concept, since these molecules are major components of specialized membrane domains called lipid rafts (Bagnat et al., 2001; Hakomori, 2003, 2008). GSLs have been characterized as important structures in cell–cell interaction, cell signaling, and protein sorting (Bagnat et al., 2000; Bagnat and Simons, 2002; Nimrichter et al., 2008; Staubach and Hanisch, 2011). Lipid rafts are more ordered and tightly packed than the surrounding bilayer, serving as organizing centers for the assembly of signaling molecules, influencing membrane fluidity and membrane protein trafficking (Chiantia and London, 2013).

The most common GSL found in fungi is GlcCer, present in the membranes of most fungi, such as *Pichia pastoris*, *C. albicans*, *Cryptococcus neoformans*, *Aspergillus fumigatus*, *Sporothrix schenckii* and *Neurospora crassa* (Saito et al., 2006). Large amounts of this GSL have also been found in the fungal cell wall (Nimrichter and Rodrigues, 2011). GlcCer has been identified as a fungal component decades ago. Its functions during fungal growth/dimorphism, lipid raft formation, and correlation with virulence have been reported (Rittershaus et al., 2006). In fact, it was recently shown to be required for virulence in *C. albicans* (de Medeiros et al., 2010; Noble et al., 2010; de Coninck et al., 2013).

Work published by Thevissen and colleagues strongly suggested that fungal GlcCer targeting by the AMPs *Rs*AFP2 and *Hs*AFP1 could initiate a cell signaling response in fungi, with formation of ROS and subsequent cell death by apoptosis (Thevissen et al., 2004; Aerts et al., 2007, 2009, 2011). The use of anti-GlcCer antibodies was shown to block germ tube formation in *C. albicans*, *Colletotrichum gloeosporioides*, and *Pseudallescheria boydii* (Pinto et al., 2002; da Silva et al., 2004), and also to protect mice upon the potentially lethal infection by *C. neoformans* (Rodrigues et al., 2007).

The crescent knowledge of GlcCer functions in eukaryotes (may these be related to virulence, growth or morphological transitions), together with the findings described above, can be connected to specific and essential structural features and particular biosynthetic steps to validate this GSL, as well as other fungal specific membrane lipids and sterols, as potential targets on the development and discovery of new antifungal drugs (Nimrichter and Rodrigues, 2011; Gonçalves et al., 2012b). Besides GlcCer, fungal membranes are also rich in phosphomannans and in the negatively charged phospholipids PS, phosphatidylinositol (PI) and diphosphatidylglycerol (DPG), which confer a highly negative surface charge to these membranes (Pasupuleti et al., 2012).

## MODELS OF MEMBRANE ACTIVITY – MECHANISM OF ACTION

The biological activity of AMPs is strongly influenced by peptide–membrane interactions. To explain how some AMPs show differential membrane affinity, their biological activities, and modes of action have been assessed on studies of defensins interaction with fungal membrane model systems, which showed a strong dependence on membrane lipid composition and on the concentration of specific components (de Medeiros et al., 2010; Gonçalves et al., 2012a,b). As with other AMPs, the mechanisms of action of some plant defensins with antifungal activity involve membrane binding, binding to the cell wall, interaction with intracellular targets leading to apoptosis, membrane permeabilization, and receptor-mediated internalization (van der Weerden et al., 2013).

The mechanisms of action of some defensins have been studied by using synthetic lipid vesicles mimicking the lipid composition of fungal, bacterial and mammal membranes (de Medeiros et al., 2010; Wimley and Hristova, 2011; Gonçalves et al., 2012a,b). The permeabilization models used to explain the mode of action of defensins could be classified into two main groups: transmembrane pore formation, such as the barrel-stave and toroidal models, and non-pore formation, such as the carpet, aggregate channel, Shai–Matsuzaki–Huang, lipid clustering, and interfacial activity models (Alba et al., 2012). The carpet model can evolve to disrupt the membrane through pore formation models or through a detergent-like mechanism, with partial micellization of the membrane (Bechinger and Lohner, 2006; Chang et al., 2008; Hoskin and Ramamoorthy, 2008). There are currently at least three different commonly accepted models describing possible AMPs mode of action: the barrel-stave pore model, the toroidal pore model, and the carpet model (Shai, 2002; Chang et al., 2008; Hoskin and Ramamoorthy, 2008; Alba et al., 2012).

**FIGURE 2:**
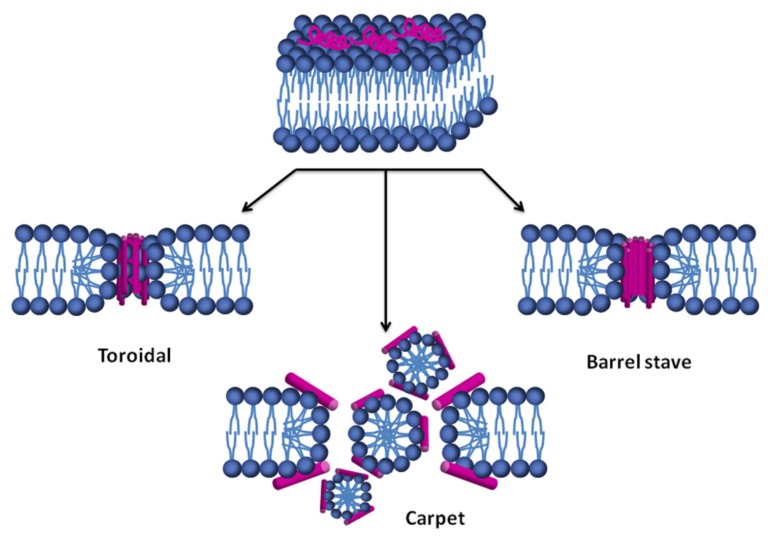
**Models of lipid membrane permeabilization by AMPs.** Initially, the peptide (magenta) is adsorbed at the membrane surface. After an initial recognition of the surface, a conformational change of the peptide occurs. Once a threshold concentration of peptide on the membrane is reached, it is followed by membrane disruption by one of these three mechanisms.

Most defensins are amphipathic molecules with clusters of positively charged amino acid residues side chains and hydrophobic amino acid side chains (Lehrer and Lu, 2011). This structural behavior allows them to interact with microbial membranes both at the level of the negatively charged phospholipid head groups and of the hydrophobic fatty acid chains. The orientation of the peptide on the membrane surface depends on the specific peptide–lipid system, but it is common for the AMP to stay at the membrane interface until a threshold peptide concentration is reached (Yang et al., 2000; Yount and Yeaman, 2005; Pasupuleti et al., 2012). In the barrel-stave model (**Figure 2**), once the critical threshold concentration of peptide is reached, peptides self-aggregate in the membrane resulting in the formation of a transmembrane pore lined by peptide, which dissipates proton and ionic gradients (Ehrenstein and Lecar, 1977; Reddy et al., 2004), but the membrane thickness and homogeneity do not change (Chang et al., 2008). The toroidal pore model is a variant of the barrel-stave model, claiming that, at some critical peptide concentration, curvature strain induces membranes to curve inward, resulting in the formation of a pore that is lined by both peptides and lipid headgroups (**Figure 2**). Toroidal pores seem to have varying lifetimes and longer-lived pores may have a lethal effect similar to barrel-stave pores, with dissipation of proton and ion gradients. This type of mechanism of AMPs action also causes a decrease in membrane thickness and a slightly decreased surface homogeneity (Chang et al., 2008). In the carpet model (**Figure 2**), peptides bind to phospholipid head groups by electrostatic interactions and align themselves parallel to the membrane surface in a carpet-like fashion until a critical threshold concentration is reached. When a detergent-like membrane micellization takes place, a strong decrease of membrane homogeneity occurs (Chang et al., 2008; Hoskin and Ramamoorthy, 2008; Epand et al., 2010; Hazlett and Wu, 2011; Li et al., 2012; Pasupuleti et al., 2012).

Besides targeting fungal membranes’ specific components, defensins may also have other mechanisms of action, as previously referred. These mechanisms comprise binding to the cell wall, membrane permeabilization, receptor-mediated internalization inducing signaling cascades and interaction with intracellular targets, which would cause the formation of ROS, leading ultimately to programed cell death. To address these mechanisms the reader is directed to some relevant references on this topic (Hancock and Rozek, 2002; Oberparleiter et al., 2003; Thevissen et al., 2003a, 2004; Schroeder et al., 2011; de Coninck et al., 2013; De Paula et al., 2013; Jaeger et al., 2013; van der Weerden et al., 2013; Zhang et al., 2013).

## CONCLUSION

The knowledge on AMPs has been increasing considerably during the last 20 years. This increased knowledge shows that AMPs have much more than only antimicrobial activity, presenting a broad spectrum of physiological functions. Defensins are the most represented AMPs across the eukaryotic domain, and in all types of eukaryotic organisms we can find defensins not only with antifungal activity but also with other potential applications.

Despite this relevance, defensins may have limitations in terms of new drug development, due to their cationic, amphiphilic, and protease labile nature, leading to a low serum half-life that limits their systemic administration (Maisetta et al., 2008). This limitation can be overcome by the use of peptidomimetics, like the substitution of natural occurring L-amino acid residues by D-amino acid residues or unusual amino acids (Oren et al., 1997; McPhee et al., 2005). Defensins bare a favorable characteristic against this problem, as their disulfide-stabilized structure confers increased protease-resistance (Wu et al., 2003). Nonetheless, defensins combine targeted antimicrobial activity with the capacity to positively modulate the immune system, and have proven to be effective across life evolution, making these peptides highly appealing as an anti-infective strategy.

Defensins have evolved as successful barrier of defense not only against bacteria, but also pathogenic fungi, present among plants, animals, and fungi. This ability may serve as a “lesson” on how selective pressures that shape organisms and their components served and continue to serve as a lever for the evolution of better defenses. Most antibiotics used nowadays are from bacterial origin or synthetic (Clardy et al., 2009; Fisher et al., 2012). The molecular design and synthesis of new molecules inspired on defensins or on other AMP structures and sequences seem to be a promising approach to develop a new and extensive field of applications, ranging from antimicrobial therapy, to their possible use as vaccine adjuvants. Therefore, a better understanding of function and mechanism of action of HDPs, specially defensins, is highly relevant for the development of new anti-infective and immunomodulatory therapeutics (Guaní-Guerra et al., 2013).

## Conflict of Interest Statement

The authors declare that the research was conducted in the absence of any commercial or financial relationships that could be construed as a potential conflict of interest.

## Footnotes

1

2

3

4
